# Timing and Protocols for Microbiome Intervention in Surgical Patients: A Literature Review of Current Evidence

**DOI:** 10.7759/cureus.86104

**Published:** 2025-06-15

**Authors:** Nicolás Idárraga Ruiz, Carlota G Herrera Giron, Carlos Alejandro Arragan Lezama, Sergio José Frias Redroban, Manuel Omar Ventura Herrera, Gustavo Adolfo Sanic Coj

**Affiliations:** 1 Surgery, Universidad de Manizales, Ibagué, COL; 2 General Practice, Facultad Mexicana de Medicina, Universidad La Salle, Mexico City, MEX; 3 General Practice, Universidad Anáhuac Xalapa, Veracruz, MEX; 4 Public Health, Pontificia Universidad Católica del Ecuador, Ambato, ECU; 5 Surgery, Centro Materno Infantil del Nordeste, Santo Domingo, DOM; 6 General Practice, Universidad San Carlos de Guatemala, Guatemala, GTM

**Keywords:** microbiome modulation, microbiota transplantation, perioperative interventions, prebiotics, probiotics, surgical recovery, synbiotics

## Abstract

Managing the gut microbiome with a personalized approach can significantly improve surgical outcomes, leading to reduced risk of infections, improved immune function, faster recovery and healing, and decreased risk of postoperative complications. This review explores microbiome-based interventions, such as probiotics, prebiotics, synbiotics, and fecal microbiota transplantation, and their roles in perioperative, preoperative, and postoperative care. Electronic databases, such as PubMed, ScienceDirect, and Google Scholar, were searched using topic-related keywords and MeSH terms. The literature search was limited to English-language peer-reviewed articles within the last 10 years, but the majority of the literature was from the last five years. Microbiome interventions have been associated with reduced postoperative complications and enhanced recovery times. The study found that changing the gut microbiome in specific ways, like using probiotics and synbiotics before and after surgery, can lead to better surgical results. For example, these treatments can lower the risk of infection at the surgery site by 40%-80% compared to standard care, help patients recover their bowel function one to two days faster, and reduce hospital stays by up to 30%. They also decrease levels of important inflammation markers like IL-6 and CRP. Using probiotics and synbiotics before surgery and continuing them for two weeks can lower infection rates and enhance recovery while managing inflammation. The beneficial effects of probiotics, prebiotics, and synbiotics support their use as effective strategies in perioperative care. However, people react differently to probiotics, prebiotics, and synbiotics because of factors like genetics, age, hormonal differences between sexes, and variations in gut microbiota based on race. Future research should focus on developing personalized microbiome-based interventions and establishing standardized protocols tailored to individual patient characteristics to enhance their effectiveness.

## Introduction and background

Gut microbes play a significant role in keeping the body healthy by controlling the immune system, maintaining metabolism and fighting off harmful bacteria and viruses [1]. Because of preoperative fasting, taking antibiotics and the effects of surgery, gut microbiota balance is often affected in surgical patients. Such a microbial change can lead to serious issues such as easier infections at a surgical site and a slower recovery process [2,3]. The majority of gut microbiota comprises bacteria, particularly from the Firmicutes and Bacteroidetes phyla, along with viruses, fungi, and archaea. These microorganisms contribute to gut barrier integrity and the production of short-chain fatty acids (SCFAs), which are essential for intestinal and systemic health [4].

One effective way to prevent and treat surgical complications is to promote or recover the good bacteria in the gut, as a connection between improper gut bacteria and difficult postoperative outcomes has been shown [5,6]. An unbalanced microbiota in the gut causes “leaky gut” and bacterial translocation into other parts of the body, increasing inflammation and making people more susceptible to infections, including sepsis and *Clostridioides difficile *overgrowth [7,8]. However, approaches like using probiotics, prebiotics, or transferring microbiota from one person, such as fecal microbiota transplantation (FMT), have the potential to reduce infections, improve the way nutrients are absorbed, and hasten recovery [9].

In a patient, before undergoing surgery, gut microbes can be affected by diet, what medications are taken, and medical conditions [10]. Normal and diverse bacteria in the gut are commonly related to positive outcomes following surgery, but dysbiosis, often seen in chronic diseases such as diabetes and inflammatory bowel disease (IBD), may increase the risk of postoperative complications [11,12]. Medications such as protein pump inhibitors (PPIs) and antibiotics, as well as low-fiber or high-fat diets, can have a significant effect on the variety of microbiomes found in the gut. It is important to be aware of these variations, since they change how patients overcome effects of surgery and related treatments [13].

Surgery can significantly alter the microbiome by causing ischemia-reperfusion injury, releasing oxidants and triggering widespread inflammation [14]. A surgical procedure, such as a colectomy, on the gastrointestinal tract often leads to microbial shifts favoring harmful bacteria over those that are helpful [15]. Besides, the use of opioids as anesthesia can slow down the gut process, resulting in bacterial overgrowth [16]. Taking antibiotics before and after surgery can lower infections; however, it also gets rid of useful bacteria along with harmful ones, which can make a second infection more likely [16].

Over time following surgery, many patients begin to show a decline in beneficial gut bacteria and more occurrences of disease-causing bacteria. High stress during surgery can disrupt the gut microbiota, increasing the risk of postoperative ileus, surgical site infections (SSIs), and systemic inflammation. Improved outcomes are associated with strategies that support microbiome recovery, such as early enteral nutrition and judicious antibiotic use to preserve beneficial bacteria. Using targeted probiotic supplements appears to bring back balance in the gut microbiome and help improve clinical results, though more research is needed to determine the specifics and timing of probiotics that offer the best effect [17].

The study aims to find out the best times to apply microbiome interventions. What are the most effective protocols for reducing the risk of SSIs and enhancing healing, and does the timing of their use influence their effectiveness? Examining recent findings based on the current literature, this review aims to provide a clear analysis of using the microbiome in surgery, which can help shape future work and guide physicians, healthcare workers, and professionals.

## Review

Methodology

This literature review explores the timing and protocols for microbiome intervention in surgical patients. A structured search of English-language, peer-reviewed articles published in the last 10 years in particular was conducted across databases including PubMed, ScienceDirect, and Google Scholar. Search terms included combinations of the following keywords: ‘Microbiome Intervention’, ‘Surgery’, ‘Pre-operative’, ‘Post-operative’, ‘Peri-operative’, ‘Prebiotics’, ‘Probiotics’, ‘Synbiotics’, ‘Fecal Microbiota Transplantation’, ‘Surgical Site Infection’, ‘Recovery’, ‘Hospital Stay’, and ‘Infection Rates’. Boolean operators (AND, OR) were used to refine and optimize search results. The authors selected articles based on their relevance and recent publication, preferably within the last 10 years. Seventeen articles were retrieved on the basis of direct relevance to the topic and review.

Optimal timings for microbiome interventions

The optimal timing for microbiome interventions depends on the clinical context. Perioperative regimens (pre- and postoperative) deliver the best overall results for elective surgeries, while preoperative-only protocols are ideal for high-risk cases [18]. Postoperative interventions are a viable fallback when preoperative planning isn’t possible, but offer limited benefits. For critically ill patients, earlier initiation is crucial [18]. Future research should focus on standardizing protocols tailored to specific surgical scenarios.

Preoperative Microbiome Interventions

Yang et al. indicated that administering probiotics, prebiotics or synbiotics in the week or two before surgery for procedures such as a liver transplant or major abdominal resections is the recommended protocol [19]. According to Flesch et al. [20], preoperative administration of beneficial microbes enhances the intestinal barrier function and modulates immune responses, thereby reducing postoperative inflammation and infection rates [20]. Grąt et al. and Kotzampassi et al. showed that patients given probiotics for more than 10 weeks ahead of liver transplant experienced 90% fewer infections following surgery [21,22]. Even a short preoperative course with synbiotics reduced IL-6 and CRP levels and also reduced the risk of infection by 85%. Preoperative intervention helps stabilize the microbiome before these interventions cause changes to it, which is very helpful [21,22].

Perioperative Microbiome Interventions

Rayes et al. found the most robust evidence supporting perioperative regimens that include about seven days of medication before and around the surgery, followed by another two weeks after the surgery [23]. The technique is useful for surgeries on the gastrointestinal tract, including colorectal and pancreatic procedures. The use of perioperative interventions prevents big changes in the microbiome during the important surgery period, both before and after the procedure. According to Flesch et al., administering synbiotics for 5 days before the surgical procedure and 14 days following the surgery dropped the number of SSIs from 21% to 2% [20]. Yang et al. reported similar success, with perioperative probiotics accelerating bowel recovery and halving diarrhea rates. The perioperative strategy works by preventing dysbiosis before it starts and supporting recovery afterward, making it the gold standard for elective surgeries [19].

Postoperative Microbiome Interventions

According to Sze et al., when preoperative preparation is not possible, starting probiotics 24-72 hours after surgery can still provide benefits, particularly for reducing ileus and late-onset infections [24]. This approach is often used in emergency surgeries or cases where planning ahead was not feasible. Bajramagic et al. found that probiotics initiated on postoperative day 3 reduced ileus rates from 23% to 2.6%. However, postoperative-only interventions are generally less effective than perioperative regimens because they miss the critical window for microbiome optimization before surgical stress occurs. While better than no intervention, their impact is limited to mitigating rather than preventing complications [25].

Special Considerations for Critically Ill Patients

In intensive care units (ICU) for critically ill patients, microbiome interventions should begin as early as possible, even if it is only after surgery. These patients often experience rapid microbiome collapse due to broad-spectrum antibiotics, sepsis, or trauma. Komatsu et al. demonstrated that early perioperative probiotics reduced major complications by 40% in high-risk cases [26]. However, McNaught et al. found that postoperative probiotics in ICU patients only modestly reduced inflammation (IL-6) without improving survival, likely because the intervention started too late. For this population, timing is even more critical, and earlier initiation yields better outcomes [27].

Strategies to reduce surgical site infections and improve postoperative recovery

Surgical Site Infections

SSIs represent a significant burden in postoperative care, contributing to prolonged hospital stays, increased healthcare costs, and delayed recovery. Sommacal et al. suggest that using treatments aimed at the microbiome, especially probiotics and synbiotics, can effectively lower the risk of SSIs and help patients recover better after surgery [28]. Synbiotics, which combine probiotics (beneficial bacteria) with prebiotics (non-digestible fibers that nourish them), have shown notable efficacy in open and high-risk surgeries. For instance, Flesch et al. demonstrated that administering synbiotics containing *Lactobacillus* and *Bifidobacterium* strains alongside fructo-oligosaccharides (FOS) led to a dramatic reduction in SSIs, from 21.4% to 2%, in patients undergoing open colorectal surgeries (p = 0.002) [20]. Similarly, Rayes et al. observed a 67% decrease in postoperative infections (12.5% vs. 40%, p = 0.005) in pancreaticoduodenectomy patients treated with synbiotics, attributing the benefit to suppression of pathogenic bacteria and enhanced mucosal immunity [23]. Furthermore, Kotzampassi et al. found that a four-strain probiotic significantly reduced SSIs (from 20% to 7.1%, p = 0.010) in colorectal surgery patients by modulating inflammatory pathways, specifically downregulating IL-6 and TNF-α via the SOCS3 mechanism [22].

Overall Recovery

Beyond infection prevention, microbiome modulation can improve postoperative recovery by restoring bowel function, reducing inflammation, and maintaining gut barrier integrity. Yang et al. showed that probiotics comprising *Bifidobacterium* and *Lactobacillus *accelerated the return of bowel function, marked by shorter time to first defecation, and halved the incidence of diarrhea in colorectal cancer patients. This effect is likely mediated by the production of short-chain fatty acids, which stimulate intestinal motility [28,19]. In line with previous study findings, Polakowski et al. reported that administering synbiotics for seven days preoperatively significantly reduced inflammatory markers such as IL-6 and CRP, correlating with fewer postoperative infections (2.8% vs. 18.9%) [29]. Additionally, Liu et al. linked the use of probiotics (*Lactobacillus* *​​​​​plantarum*, *Bifidobacterium longum*) to improved gut barrier function, as evidenced by lower serum zonulin levels, a marker of intestinal permeability, and a 30% reduction in postoperative infections in patients undergoing liver metastasis surgery (p = 0.008) [30].

Rayes et al. recommended that clinicians begin giving synbiotics or probiotics to patients one to two weeks in advance of high-risk surgery involving high-risk scopes [31]. Deng et al. indicated that blends combining *Lactobacillus*,* Bifidobacterium *and FOS are preferred to maximize both their effects and how well the bacteria stay alive in the gut. Checking zonulin and IL-6 biomarkers is another way to choose the right treatment for each patient [32]. Using strategies that target the microbiome in the perioperative period seems to assist in reducing SSIs and promoting a quicker recovery. Synbiotics have been found to decrease infections by up to two-thirds, compared to probiotics by improving bowel function, reducing whole-body inflammation and strengthening the gut barrier. These interventions as part of surgery allow healthcare professionals to greatly boost outcomes and limit postoperative risks.

Different microbiome intervention approaches

Probiotics, Preoperative Administration

Bajramagic et al. has demonstrated that taking probiotics before surgery increases immune health and maintains digestive function. The administration of probiotics right before surgery encourages helpful bacteria, leading to fewer cases of postoperative infections [25]. According to Kotzampassi et al., major complications were seen much less frequently when people received probiotics a day before surgery and for 15 days more afterward (28.6% vs. 48.8%) [22]. According to Polakowski et al., current evidence suggests that using symbiotics for one week before an operation lowers the IL-6 and CRP markers and leads to fewer infection problems (2.8% in comparison to 18.9%). The results suggest that taking probiotics helps the gut to manage stress before surgery [29].

Probiotics, Perioperative Administration

The perioperative administration of probiotics shows that giving them before and after surgery is the most beneficial. This way of preparing lets the surgeons take care of the patient's microbiome, which leads to better healing after surgery and a stronger immune system. Rayes et al. reported that pre-surgery probiotic use for seven days (starting one day before surgery) reduced the risk of diarrhea (26.67% chance) and improved bowel function when compared to those who were given no probiotics [33]. Park et al. found that probiotics taken prior to surgery shifted the beneficial bacteria in the gut and lowered disease-causing inflammation. The research proves that probiotic treatment planned for weeks before and after surgery is beneficial for each stage of the procedure [34].

Probiotics, Postoperative Administration Only

The benefits of administering probiotics postoperatively appear to be less pronounced compared to pre- or perioperative administration. Postoperative factors such as antibiotic use and surgical stress can significantly disrupt gut microbiota, making it more challenging for administered probiotics to colonize and exert their effects. In a study by Grąt et al. and Bajramagic et al. [21,25], probiotics administered three days postoperatively were associated with a significant reduction in postoperative ileus (2.6% vs. 23.1%). However, no significant improvements were observed in other major postoperative complications, suggesting that postoperative administration alone may be insufficient to counteract surgery-induced dysbiosis. Therefore, the exclusive use of postoperative probiotics should be considered as an adjunctive strategy rather than a primary intervention [21,25].

Synbiotics, Perioperative Administration

The perioperative use of both probiotics and prebiotics offers a strong strategy because it helps in promoting the growth and health of good microbes. McNaught et al. indicated that the combined use of both drugs has been proven to reduce surgical infections, mostly in more challenging surgical cases. Their study showed that using synbiotics for five days prior to surgery and 14 days afterwards reduced infections from 21.4% to 2% [27]. Rayes et al. showed that giving synbiotics before and after pancreaticoduodenectomy reduced the risk of infectious complications (from 40% to 12.5%). These data suggest that the ongoing modification of microbes and nutrition in the gut helps improve outcomes after surgery [33].

Synbiotics, Preoperative-Only Administration

Taking synbiotics before surgery eases inflammation but may fail to provide all the postoperative benefits. Deng et al. found that microbiotas may be handy before surgery to help the body fight infection and reduce recovery stress; administering them after the operation offers no significant support [32]. In the study by Polakowski et al., taking synbiotics before surgery for seven days improved the early results and reduced some inflammation markers, but there was no follow-up data on extra recovery periods or infections. Hence, it appears that using preoperative synbiotics is helpful, but they are most successful when patients continue using them after surgery [29].

Prebiotics (Fiber-Based)

The study by Sze et al. focused on prebiotics, which are essential for growing helpful bacteria. Prebiotics are commonly included in synbiotic mixtures in clinical trials, which prevents researchers from studying them alone [24]. These types of prebiotics, made from fiber, tend to be included in normal enteral nutrition strategies and not exclusively in microbiome-focused approaches. Therefore, high-quality research on prebiotics and surgery apart from other treatments is lacking [24]. Further studies are required to understand how much prebiotics affect gut health and the outcomes following surgery.

Fecal Microbiota Transplantation

FMT involves the transfer of stool from a healthy donor to a recipient to restore a disrupted gut microbiome and is well-established in the treatment of recurrent *C. difficile* infections. FMT is primarily used in chronic dysbiosis rather than acute, surgery-induced disturbances [28]. Moreover, the logistics, regulatory constraints, and patient acceptance of FMT in perioperative care present challenges [28]. Nevertheless, its potential in severe dysbiosis scenarios remains an area worth exploring in future trials.

Intervention-Specific Effects

Different microbiome strategies offer distinct advantages depending on their composition and timing. Rayes et al. indicated that probiotics primarily enhance bowel function and immune regulation, making them ideal for improving gastrointestinal recovery [31]. Synbiotics, due to the synergistic effect of combining probiotics and prebiotics, improve prevention of infections, especially in complex or high-risk surgeries. Flesch et al. concluded that the dual mechanism of action enhances colonization and resilience of beneficial microbes, which is particularly important in surgical patients exposed to antibiotics and physiological stress [20].

Clinical Efficacy

Prebiotics, probiotics and synbiotics have been found to help improve surgical patient results in many clinical studies. As a result of these treatments, fewer postoperative infections occur since pathogenic bacteria are blocked, gut function improves and the body’s immune reaction is managed. Flesch et al. indicated that synbiotics reduced SSIs from 21.4% to 2% in colorectal cancer patients, and according to Sommacal et al., overall infections were reduced from 69.6% to 26% for surgeries on periampullary neoplasm tumors [20,28].

Yang et al., Kotzampassi et al., and Polakowski et al. suggested that probiotics help surgery patients by restoring gut movement, bringing on bowel movements sooner, and shortening recovery time. Using probiotics appeared to greatly lower the risk of major complications such as leakage and pneumonia [19,22,29]. Park et al. suggested that therapies for the microbiome help create beneficial changes in bacteria, which is useful against dysbiosis from surgery and antibiotic use. A study found that probiotics helped grow *Akkermansia*, a helpful anti-inflammatory bacteria, but reduced pathogenic bacteria [34].

According to Polakowski et al., Grąt et al., and Liu et al., these therapies not only help keep systemic inflammation lower, as evidenced by decreases in IL-6 and CRP, but also enhance gut health by lowering zonulin levels. The use of probiotics before surgery reduced post-transplant infections by 30% in cases where liver transplantation was involved [21,29,30]. Kotzampassi et al. and Fang et al. concluded that when the timing of intervention is examined, the best effects come from timing the therapy both before and after surgery [22,35]. Issues such as working with a few samples, strain-related changes and antibiotics still need to be overcome. All microbiome interventions should be further validated in several large, long-term trials that follow standard protocols. All things considered, microbiome treatments seem to be a good and antibiotic-free way to improve how patients recover from surgery and keep complications low. Bajramagic et al. indicated that in addition to protecting against infection, changing the microbiome in the body can minimize the risk of serious problems after surgery. In another case, probiotics decreased ileus (from 23.1% to 2.6%) in those with colorectal cancer, making it clear that probiotics can restore gut function [25]. Colorectal liver metastasis patients given probiotics experienced fewer cases of septicemia (59% less frequently) and urinary tract infections (13% fewer). Such interventions help maintain healthy levels of gut bacteria, which can be changed by bowel preparation and antibiotics after surgery. Sze et al. revealed that microbiota in colorectal cancer patients after treatment resembled those found in healthy people, suggesting the microbiome helps speed recovery [24]. Moreover, Polakowski et al. found synbiotics decreased inflammatory substances, such as IL-6 and CRP, promoting wound healing [29].

Rayes et al. found that symbiotics cut infections by over 50% in pancreaticoduodenectomy; Fang et al. saw that patients undergoing colectomy continued to experience microbiome problems, calling for microbial support after surgery [33,35]. The research study by Liu et al. revealed that probiotics help the liver regain its normal function more quickly after hepatobiliary surgery [30], but some trials do not produce the same results. Komatsu et al. discovered that SSI reduced in colorectal surgery, except in laparoscopic surgery, because of differences in approaches [26]. Similarly, McNaught et al. found that using probiotics in the ICU did not result in a reduction in patient mortality [27]. Variables and outcomes for the 17 clinical studies included in this review are described in Table 1.

**Table 1:** Characteristics of studies included in the review CRC: colorectal cancer, TID: three times a day, SSI: surgical site infection, FOS: fructo-oligosaccharide, CFU: colony-forming unit, IL-6: interleukin 6, TNF-α: tumor necrosis factor alpha, SOCS3: suppressor of cytokine signaling 3, MELD: Model for End-Stage Liver Disease, CTP: Child-Turcotte-Pugh (score for liver disease), ALT: alanine aminotransferase, AST: aspartate aminotransferase, INR: international normalized ratio (for blood clotting), BID: twice a day, qPCR: quantitative polymerase chain reaction, HPLC: high-performance liquid chromatography, RT-qPCR: reverse transcription quantitative polymerase chain reaction, 16S rRNA: ribosomal RNA (used in microbial community analysis), PCR: polymerase chain reaction, BMI: body mass index, IBD: inflammatory bowel disease, IBS: irritable bowel syndrome, EAD: early allograft dysfunction, MAPK: mitogen-activated protein kinase, QIIME: Quantitative Insights Into Microbial Ecology (bioinformatics tool), LEfSe: Linear Discriminant Analysis Effect Size (bioinformatics tool), LDA: linear discriminant analysis, FMT: fecal microbiota transplantation, NLR: neutrophil-to-lymphocyte ratio; CRP: C-reactive protein (inflammation marker), LC-MS/MS: liquid chromatography-tandem mass spectrometry; PPPD: pancreaticoduodenectomy, LT: liver transplantation, CLM: colorectal liver metastases; LoS: length of stay

Author	Study design/population	Objectives	Types of intervention	Timing for microbiome intervention	Protocols for microbiome intervention	Outcome measured	Study findings	Overall conclusion	Challenges
Yang et al. [19]	Randomized controlled trial: 60 patients with confined colorectal cancer undergoing resection	To evaluate anti-infective effects of perioperative probiotics in CRC patients	Combined oral probiotics: *B. longum*, *L. acidophilus*, *E. faecalis*	5 days before to 7 days after surgery (12 days total)	2g probiotics orally TID (or gastric gavage post-op Day 1); Bifico (China); blinded design; combined with standard bowel prep and prophylactic antibiotics	Days to first flatus/defecation, fluid/solid diet, pyrexia, drainage duration, infection rates, hospital stay, blood indices	Earlier return of bowel function; lower diarrhea incidence (26.67% vs. 53.33%); slightly lower bacteremia (not statistically significant); no mortality or major adverse effects	Probiotics improved bowel recovery and reduced some short-term complications; clinically valuable in CRC surgery	Short intervention duration; no post-discharge probiotic continuation; unclear long-term benefits
Flesch et al. [20]	Randomized, double-blind, placebo-controlled trial; 91 colorectal cancer surgery patients	To assess the impact of perioperative synbiotics on infection rates	Synbiotics: *L. acidophilus, L. rhamnosus, L. paracasei, B. lactis* + FOS	5 days before and 14 days after colorectal cancer surgery	2 sachets/day, twice daily; sachets contained probiotics (10⁸-10⁹ CFU each) + 6g FOS (intervention) or 96% maltodextrin (placebo); administered orally; routine bowel prep and antibiotics given to all patients one hour pre-surgery	Incidence of surgical site infection, intra-abdominal abscess, pneumonia, other infections, hospital stay	SSI in 2% of symbiotic group vs. 21.4% in control (p=0.002); 0 vs. 7 cases of other infections (p=0.001); no difference in non-infectious complications or length of stay; all infections occurred in open surgery patients	Perioperative oral synbiotics significantly reduced postoperative infections in CRC surgery.	No difference in hospitalization time; effect observed only in open (not laparoscopic) surgeries; product-specific effects may not generalize; no long-term follow-up
Rayes et al. [23]	Review of 15 RCTs and animal studies involving surgical patients (pancreatic, liver, colorectal, trauma, transplant)	To assess the efficacy and safety of pre-, pro-, and synbiotics in surgical patients for preventing infections	Prebiotics (fibers), probiotics (e.g., *Lactobacillus, Bifidobacterium*), synbiotics (combinations, e.g., Synbiotic 2000, BIO-THREE, Trevis)	Varied: pre-op (3-15 days), post-op (up to 28 days); perioperative regimens tested	Various regimens: some oral, some enteral (nasojejunal); examples: Synbiotic 2000 (multiple *Lactobacillus *strains + beta-glucan, inulin, starch, pectin); BIO-THREE (*Enterococcus, C. butyricum, B. mesentericus*); duration 5-28 days; different combinations tested across patient types	Infection rate, hospital stay, inflammatory markers, mortality, immune response	Synbiotics reduced infections in 10/15 RCTs; some benefit in liver/pancreas/trauma patients; no serious side effects in most; one trial showed increased mortality (16% vs. 6%) in severe acute pancreatitis (possible ischemia from fiber load)	Synbiotics are effective in high-risk surgical patients (liver, pancreas, trauma); effect depends on strain, dose, and duration.	Variable results due to heterogeneity in products, durations, and patient risk profiles; short post-op use, oral (vs. enteral) route, and inconsistent probiotic survival impacted efficacy; caution in critically ill (risk of bowel ischemia in one trial)
Sze et al. [24]	Prospective cohort: 67 patients (22 adenoma, 19 advanced adenomas, 26 carcinoma) with pre- and post-treatment fecal samples	To assess if treatment for colonic lesions shifts gut microbiota composition toward that of healthy individuals	Polyp removal, surgical resection, chemotherapy, radiation	Fecal samples collected before treatment and 188-546 days after treatment	16S rRNA gene sequencing of V4 region from stool samples; microbial analysis through diversity metrics and random forest modeling	Changes in microbial community structure; resemblance to normal microbiota; probability scores from diagnostic models	Carcinoma patients had significant microbiota shifts toward normal post-treatment	Patients with carcinoma showed notable changes in their flora toward normal post-treatment.	Not reported
Komatsu et al. [26]	Single-center randomized controlled trial; 362 patients undergoing laparoscopic colorectal surgery (168 synbiotics, 194 control)	To assess whether perioperative synbiotics reduce SSI and alter fecal microbiota	Synbiotics: Yakult Ace (*L. casei* *Shirota* + galacto-oligosaccharides) and MILMIL-S (*B. breve *Yakult)	7-11 days pre-op and 2-7 days post-op	Oral synbiotics daily; microflora analyzed by RT-qPCR (YIF-SCAN®), fecal organic acids by HPLC	Primary: SSI within 30 days. Secondary: microbiota changes, organic acid levels, fecal pH	No significant reduction in SSI (17.3% synbiotics vs. 22.7% control, p=0.2)	Symbiotic use did not lead to a significant reduction in surgical site infections compared to the control group.	Not reported
Sommacal et al. [28]	Randomized, double-blind clinical trial; 46 patients undergoing surgery for periampullary neoplasms (23 synbiotics, 23 placebo)	To evaluate whether synbiotics reduce postoperative complications and mortality	Synbiotics: *L. acidophilus, L. rhamnosus, L. casei, B. bifidum*, and FOS	4 days before to 10 days after surgery (14-day course)	Oral capsules administered twice daily; placebo-controlled; standardized dosing and storage	Post-op complications, infection rate, antibiotic use, hospital stay, mortality, nutritional status	Infections: 26% (synbiotics) vs. 69.6% (control), p=0.008	The use of synbiotics significantly reduced infection rates compared to the control group.	Not reported
Rayes et al. [31]	Prospective, randomized, placebo-controlled trial; 90 patients undergoing major abdominal surgery (liver, stomach, colon, pancreas resections)	To compare early enteral nutrition (with fiber and *Lactobacillus*) vs. conventional parenteral nutrition for reducing postoperative infections	Group A: parenteral/fiber-free enteral nutrition; Group B: fiber + live *Lactobacillus*;* *Group C: fiber + heat-killed *Lactobacillus*	Early postoperative period (initiated shortly after surgery)	Enteral nutrition via fiber-containing formulas: Group B: live *Lactobacillus*; Group C: heat-killed *Lactobacillus*;* *Group A: conventional nutrition (control)	Incidence of infections, duration of antibiotics, hospital stay, bowel movement onset, immune/nutritional parameters	Infection rate significantly lower in Groups B and C (10%) vs. Group A (30%) (p=0.01); antibiotic duration significantly shorter in Group B (p=0.04); no significant difference in hospital stay or non-infectious complications; benefits of live *Lactobacillus* seen in the gastric/pancreatic surgery subgroup	Early enteral nutrition with fiber reduces infection risk. Live *Lactobacillus *may offer additional benefits in certain surgery types.	Small subgroup sizes limited statistical analysis (e.g., gastric/pancreatic cases). No blinding mentioned. Lack of microbiome composition analysis. Generalizability may be limited due to specific surgical cohort
Deng et al. [32]	Original research using 16S rRNA sequencing; 69 total subjects: 33 healthy, 17 CRC untreated, 14 chemo-treated, 5 surgery-treated	To assess how anti-cancer treatments (surgery or chemotherapy) affect gut microbiota and identify potential microbial biomarkers for CRC therapy	Anti-cancer interventions: surgery or chemotherapy (oxaliplatin + tegafur)	Post-treatment sampling (after surgery or chemotherapy)	16S rRNA sequencing of fecal samples; analysis via QIIME, LEfSe/LDA scoring for biomarker detection	Microbial richness (Chao1), diversity (Shannon), taxonomic profiles, and potential biomarkers at multiple taxonomic levels	Surgery reduced microbial diversity. *Fusobacterium *enriched in untreated and chemo-treated CRC patients. Proteobacteria enriched after surgery. *Veillonella dispar* and *Sutterella* are linked to chemotherapy.	Microbiota shifts significantly with treatment type; potential microbial biomarkers could improve CRC treatment and monitoring.	Small sample size in the surgical group, observational design, no functional validation of biomarkers, no actual microbiota-modulating intervention used
Park et al. [34]	RCT; 60 patients undergoing anterior resection for sigmoid colon cancer (29 probiotics, 31 placebo)	To evaluate probiotic effects on microbiota, recovery, and inflammation post-colon resection	Multi-strain probiotics (*B. animalis *HY8002, *L. casei* HY2782, *L. plantarum* HY7712) vs. placebo (prebiotics only)	1 week pre-op to 3 weeks post-op (4 weeks total)	2g twice daily; mechanical bowel prep + ciprofloxacin/metronidazole; fecal samples at baseline, pre-op, 3- and 4-weeks post-op	Primary: ARS scores. Secondary: microbiota composition, inflammatory markers (zonulin, WBC, NLR), complications, QoL	Increase in* Bifidobacterium*,* Akkermansia*;* *decrease in *Alloprevotella*,* Porphyromonas*; better flatus control (p=0.030), lower zonulin (p=0.035), fewer complications (6% vs. 28.5%, p=0.024); *Bifidobacterium *inversely correlated with zonulin	Probiotics helped modify microbiota, reduce inflammation, and improve bowel function and surgical recovery.	Short follow-up: placebo contained prebiotics; small sample size reduced power for complication-related outcomes
Kotzampassi et al. [22]	Randomized, double-blind, placebo-controlled trial; 164 patients undergoing elective open colorectal surgery (84 probiotic, 80 placebo)	To assess the effect of a 4-strain probiotic regimen on postoperative complications and immune modulation	*L. acidophilus* LA-5, *L. plantarum, B. lactis *BB-12, *Saccharomyces boulardii *vs. placebo (glucose polymer)	Started 1 day before surgery (after bowel prep) and continued for 15 days postoperatively	Dosage: initial loading dose (4 capsules pre-op), then 1 capsule twice daily. Bowel prep: mechanical bowel cleansing preoperatively. Administration: via drinking water or nasogastric tube if intubated	Primary: major postoperative complications (infections, anastomotic leakage, mechanical ventilation). Secondary: time to bowel movement/defecation, hospital stay, cytokine levels (IL-6, TNF-α), and SOCS3 gene expression	Complications: the probiotic group had lower rates of major complications (28.6% vs. 48.8%, p=0.010), pneumonia (2.4% vs. 11.3%), SSIs (7.1% vs. 20%), and anastomotic leakage (1.2% vs. 8.8%). Recovery: shorter time to bowel movement (p<0.0001) and hospital discharge (8 vs. 10 days). Immune modulation: probiotics correlated with SOCS3-mediated regulation of TNF and IL-6 (p=0.004).	Probiotic prophylaxis significantly reduced postoperative complications and accelerated recovery, likely via immune modulation (SOCS3 pathway).	The study stopped prematurely due to efficacy, limiting long-term data. Traditional bowel prep and open surgery may not reflect modern practices (e.g., laparoscopy). Placebo contained a glucose polymer, which may mildly affect gut microbiota.
Bajramagic et al. [25]	Randomized controlled prospective study; 78 patients with colorectal adenocarcinoma (39 probiotic, 39 placebo)	To evaluate the clinical significance of probiotics in reducing postoperative complications and improving outcomes in colorectal cancer surgery	8-strain probiotic capsule (*Lactobacillus acidophilus*, *L. casei, L. plantarum, L. rhamnosus, Bifidobacterium lactis, B. bifidum, B. breve, Streptococcus thermophilus*) vs. no probiotics	Started on postoperative day 3, continued for 30 days (2 capsules/day), then 1 capsule/day for 2 weeks/month up to 1 year	Dosage: 2 capsules/day for 30 days, then 1 capsule/day intermittently. Administration: oral, with liquid diet initiation post-op. Bowel prep: standard preoperative protocols (not detailed)	Primary: postoperative complications (ileus, SSI, anastomotic leakage, intra-abdominal abscess). Secondary: mortality, hospital stay duration, tumor localization-specific outcomes	Complications: significant reduction in ileus (2.6% vs. 23.1%, p=0.007). Non-significant trends favoring probiotics for SSIs (28.2% vs. 35.9%) and anastomotic leakage (5.1% vs. 12.8%). Mortality: lower 6-month mortality in the probiotic group (0% vs. 2.6%, p>0.05). Hospital stay: shorter in the probiotic group (p<0.05). Tumor localization: greatest reduction in complications for rectal (-33.3%) and ascending colon (-16.7%) tumors	Probiotics significantly reduced ileus and hospital stay, with potential benefits for SSI and mortality. The strongest effects were observed in rectal/ascending colon tumors.	Small sample size limited statistical power for some outcomes. A long-term probiotic regimen (1 year) may affect compliance. Lack of detailed microbiome analysis to link clinical outcomes to microbial changes
Liu et al. [30]	Double-center, double-blind randomized clinical trial of 150 patients with CLM. The final analysis included 117 patients (66 in the probiotics group and 68 in the control group).	To investigate the effects of perioperative probiotics on serum zonulin (intestinal permeability marker), liver barrier function, and postoperative infectious complications after CLM surgery	Probiotic mixture (*Lactobacillus plantarum, L. acidophilus, Bifidobacterium longum*; total dose: 2.6 × 10^14^ CFU/day) vs. placebo (maltodextrin), administered orally for 6 days preoperatively and 10 days postoperatively	Preoperative: 6 days before surgery. Postoperative: 10 days after surgery	Protocols for microbiome intervention: probiotic formulation, acid-resistant capsules containing 3 strains. Dosage: 2 g/day (total 2.6 × 10^14^ CFU/day). Standardized care: bowel preparation, antibiotics (ceftriaxone + metronidazole), and diet control	Primary: serum zonulin levels, plasma endotoxin, postoperative infectious complications (septicemia, urinary/diarrheal infections). Secondary: liver function (ALT/AST), hospital stay duration, antibiotic use, p38 MAPK pathway activity	Reduced infections: probiotics lowered septicemia (59% vs. 88%, p=0.008), urinary infections (2% vs. 13%, p=0.017), and diarrhea (24% vs. 46%, p=0.012). Biomarkers: decreased serum zonulin (0.42 vs. 1.36 ng/mg, p<0.001) and endotoxin (p<0.001). Liver function: improved ALT/AST levels (p<0.05). Hospital outcomes: shorter pyrexia duration (6.02 vs. 6.98 days, p=0.006) and hospital stay (11.26 vs. 12.96 days, p<0.001)	Perioperative probiotics reduced zonulin levels, stabilized liver/intestinal barriers, and decreased infectious complications in CLM patients. Proposed a "clinical regulatory pathway" linking probiotics to improved outcomes via p38 MAPK inhibition.	Confounding: standard perioperative antibiotics may influence microbiome outcomes. Generalizability: limited to CLM patients; results may not apply to other surgeries. Sample size: subgroup analyses (e.g., high vs. low zonulin) had small samples.
Fang et al. [35]	Prospective cohort study of 129 IBD patients (50 ulcerative colitis, 79 Crohn’s disease) with 332 stool samples collected longitudinally. Subgroups included patients with ileocolonic resection (n=21), colectomy (n=17), and no surgery (n=91).	To characterize microbiome and metabolome changes after different surgeries for IBD (ileocolonic resection, colectomy) and compare with nonsurgical patients	Surgical interventions (ileocolonic resection, colectomy) as irreversible treatments for IBD. No direct microbiome interventions (e.g., probiotics, FMT) were tested.	Samples were collected up to 24 months post-surgery, with longitudinal sampling every ~6 months. Surgeries occurred a median of 3 years (IQR: 1-5.5 years) before baseline stool collection.	Standard perioperative antibiotics were administered during surgeries	Microbiome diversity (alpha/beta diversity via shotgun metagenomics). Metabolome diversity (untargeted LC-MS/MS metabolomics). Specific taxa/metabolite shifts (e.g., *E. coli*, bile acids). Microbiome instability over time	Diversity: surgery (especially colectomy) persistently reduced microbiome/metabolome alpha diversity (p=7.09e-16). Instability: microbiome (but not metabolome) became more unstable post-surgery. Taxa: *E. coli *expanded post-surgery; butyrate producers (e.g., *Faecalibacterium prausnitzii*) declined. Metabolites: primary bile acids increased post-surgery; secondary bile acids showed no significant change. Classification: microbiome data better predicted surgery status (average precision 0.80) than metabolomics (0.68).	Intestinal surgery in IBD patients leads to long-term reductions in microbiome/metabolome diversity and increased microbiome instability, with colectomy having the strongest effect. These changes may have clinical implications for disease management and microbiome-targeted therapies.	Confounding by antibiotics (routinely given during surgery). Heterogeneity in disease severity, surgery types, and medical therapies. Lack of pre-surgery microbiome data for most patients. Small sample sizes for specific surgery subgroups (e.g., colectomy with ileostomy)
Rayes et al. [33]	Double-blind, randomized controlled trial of 80 patients undergoing pylorus-preserving pancreaticoduodenectomy (PPPD). Excluded 9 patients due to unresectable tumors; the final analysis included 40 patients in the symbiotic group and 40 in the fiber-only (control) group.	To evaluate whether a symbiotic combination (probiotics + prebiotics) reduces postoperative bacterial infections compared to fibers alone in PPPD patients	Symbiotic group (A): 4 probiotic strains (*Lactobacillus paracasei*,* L. plantarum*, *Pediococcus pentosaceus*, *and* *Leuconostoc mesenteroides*) + 4 prebiotics (beta-glucan, inulin, pectin, and resistant starch). Control group (B): fibers only (no probiotics)	Preoperative: started 1 day before surgery. Postoperative: continued for 8 days post-surgery	Symbiotic group (A): 4 probiotic strains (*Lactobacillus paracasei*,* L. plantarum*,* Pediococcus pentosaceus*,* and Leuconostoc mesenteroides*) + 4 prebiotics (betaglucan, inulin, pectin, and resistant starch). Control group (B): fibers only (no probiotics)	Preoperative: started 1 day before surgery. Postoperative: continued for 8 days post-surgery	Infections: the symbiotic group had significantly lower infection rates (12.5% vs. 40%, p=0.005). Antibiotic use: shorter duration in the symbiotic group (2 vs. 10 days, p=0.015). Hospital stays: trend toward shorter stays (17 vs. 22 days). Safety: no adverse effects from symbiotics	Early enteral nutrition with symbiotics significantly reduced postoperative infections and antibiotic use in PPPD patients, suggesting gut microbiome modulation as a protective strategy.	Generalizability: single-center study with strict inclusion criteria. Confounding: all patients received standard antibiotics, which may influence microbiome outcomes. Sample size: underpowered for subgroup analyses (e.g., infection types)
Grąt et al. [21]	Randomized, double-blind, placebo-controlled trial; 55 adult cirrhotic patients listed for LT	To evaluate the effects of continuous preoperative probiotic administration on pre- and post-transplant outcomes, including infection rates, mortality, and graft function	Probiotic capsules (ProBacti 4 Enteric®) containing *Lactococcus lactis *(50%),* Lactobacillus* *casei *(25%), Lactobacillus* acidophilus* (12.5%), and *Bifidobacterium bifidum* (12.5%) vs. placebo	Daily administration from enrollment until LT (median duration: 45.8% >10 weeks, 37.5% 2-10 weeks, 16.7% <2 weeks)	Oral intake once daily before breakfast. No postoperative administration. Routine perioperative antibiotics (piperacillin/tazobactam + fluconazole) for all patients	Primary: 90-day postoperative mortality. 30-day postoperative infection rate. Secondary: early allograft dysfunction (EAD), bilirubin, AST/ALT, INR. Pre-transplant MELD/CTP scores, fecal microbiota changes	Infection rates: 30-day: 4.8% (probiotic) vs. 34.8% (placebo), p=0.02. 90-day: 4.8% vs. 47.8%, p=0.002. Graft function: lower bilirubin (p=0.02) and faster AST/ALT decline (p=0.03) in the probiotic group. Mortality: no deaths in the probiotic group vs. 4.3% in the placebo group (p>0.99). Microbiota: increased *Bacteroides *spp. (p=0.008) and *Enterococcus *spp. (p=0.04) in the probiotic group	Continuous preoperative probiotics reduced post-LT infections and improved early graft function but did not affect mortality or pre-transplant outcomes.	High refusal rate (administrative barriers). Small sample size (underpowered for mortality analysis). No postoperative probiotic continuation. Culture-based microbiota analysis (limited scope)
Polakowski et al. [29]	Prospective, randomized, double-blind, placebo-controlled study; 73 patients with colorectal cancer undergoing colorectal resection	To assess the effect of preoperative symbiotic administration on inflammatory response, postoperative complications, and clinical outcomes in colorectal cancer patients	Symbiotics (probiotics: *Lactobacillus acidophilus, L. rhamnosus, L. casei, Bifidobacteria lactis*; prebiotic: fructooligosaccharide) vs. placebo (maltodextrin).	7 days preoperatively (last dose administered the day before surgery)	Patients ingested symbiotics or a placebo diluted in 100 mL of water twice daily for 7 days. No postoperative administration	Inflammatory markers (IL-6, CRP), postoperative infectious/non-infectious complications, antibiotic usage, hospital LoS, and mortality	Significant reduction in IL-6 (163.2 → 138.8 pg/mL) and CRP (10 → 7.17 mg/dL) in the symbiotic group (p<0.001). Lower infectious complications (2.8% vs. 18.9%, p=0.02) and antibiotic usage (1.42 vs. 3.74 days, p<0.001) in the symbiotic group. Shorter hospital LoS (3 vs. 4 days, p<0.001). No deaths in the symbiotic group vs. 3 deaths in the placebo group (p=0.115).	Preoperative symbiotic administration attenuates inflammation, reduces postoperative morbidity, and improves clinical outcomes in colorectal cancer surgery.	Single-center study. No microbiologic evaluation of gut flora changes. Limited to preoperative intervention; postoperative effects unclear. Small sample size for certain subgroups (e.g., stage III patients)
McNaught et al. [27]	Randomized, double-blind, placebo-controlled trial; 103 critically ill patients admitted to the ICU	To investigate the effect of *L. plantarum* 299v on gut barrier function, systemic inflammation, and clinical outcomes in critically ill patients	Probiotic group: Oral L. plantarum 299v (ProViva drink, ~1 × 10^10^ CFU/day) + conventional therapy. Control group: conventional therapy alone.	Probiotic group: oral *L. plantarum *299v (ProViva drink, ~1 × 10^10^ CFU/day) + conventional therapy. Control group: conventional therapy alone	Dose: 500 mL/day target (median intake: 213 mL/day). Delivery: oral or via nasogastric tube (continuous or bolus). Concomitant therapy: antibiotics, inotropes, and nutrition as needed	Primary: gastric colonization, intestinal permeability (lactulose/rhamnose ratio), and endotoxin exposure (IgM EndoCAb). Secondary: systemic inflammation (CRP, IL-6), septic morbidity, and mortality	Gut barrier: no significant differences in gastric colonization, permeability, or endotoxin exposure. Inflammation: lower IL-6 levels in the probiotic group at day 15 (p=0.04). No CRP differences. Clinical outcomes: no reduction in septic complications (40% vs. 43%) or mortality (35% in both groups)	*L. plantarum* 299v attenuated systemic inflammation (reduced IL-6) but did not improve gut barrier function or clinical outcomes in critically ill patients. Routine use not supported	Low probiotic detection in gastric aspirates (26% at day 4). High antibiotic use may have masked probiotic effects. Underpowered for mortality/sepsis outcomes. Practical issues with enteral delivery in critically ill patients

Clinical implications

From a clinical standpoint, early and sustained microbiome modulation is essential for achieving meaningful health outcomes. Starting probiotics or synbiotics preoperatively allows time for colonization, while continuing them postoperatively maintains microbial stability during a vulnerable period. In contrast, initiating treatment only after surgery may be less effective due to a compromised gut environment. These insights emphasize the need for optimized timing of microbiome therapies in surgical care for infection prevention, inflammation control, and patient recovery.

Challenges and limitations

Not all studies reported positive outcomes. Komatsu et al. found no reduction in SSIs with synbiotics during laparoscopic surgery [26]. McNaught et al. found no reduction in mortality among ICU patients [27]. Variability in probiotic strains, high antibiotic use, and patient population differences can influence results. Several studies had the limitations of small sample sizes, limited follow-up durations, and significant heterogeneity in strains, dosages, and timing. These issues make it difficult to draw definitive conclusions.

Future direction

Future research should prioritize the development of personalized probiotic therapies tailored to individual microbiome profiles. This approach involves customizing probiotic interventions based on a person's unique gut bacteria to enhance effectiveness. Combining probiotics with prebiotics or postbiotics may further improve outcomes by supporting and sustaining beneficial microbial activity. Additionally, establishing standardized treatment protocols, including specific strain selection, appropriate dosage, and optimal timing, will be essential for ensuring consistent and effective results across diverse populations.

## Conclusions

Microbiome-based interventions have shown substantial promise in surgical settings by reducing infections, enhancing recovery, and controlling inflammation. These benefits highlight the potential of probiotics, prebiotics, and synbiotics as supportive strategies in perioperative care. However, the widespread adoption of such interventions requires rigorous, statistically powered clinical trials. Standardized protocols need to be established, and long-term outcomes must be carefully assessed to ensure safety, consistency, and efficacy across diverse patient populations. Therefore, effective intervention design requires interdisciplinary collaboration among surgeons, microbiologists, dietitians, and nutritionists.

## References

[1] Deng J, Huang Y, Yu K, Luo H, Zhou D, Li D (2024). Changes in the gut microbiome of patients with esophageal cancer: a systematic review and meta-analysis based on 16S gene sequencing technology. Microb Pathog.

[2] Derosa L, Iebba V, Silva CA (2024). Custom scoring based on ecological topology of gut microbiota associated with cancer immunotherapy outcome. Cell.

[3] Fonnes S, Mollerup S, Paulsen SJ, Holzknecht BJ, Westh H, Rosenberg J (2024). The microbiome of the appendix differs in patients with and without appendicitis: a prospective cohort study. Surgery.

[4] Fujimoto K, Hayashi T, Yamamoto M (2024). An enterococcal phage-derived enzyme suppresses graft-versus-host disease. Nature.

[5] Glitza IC, Seo YD, Spencer CN (2024). Randomized placebo-controlled, biomarker-stratified phase Ib microbiome modulation in melanoma: impact of antibiotic preconditioning on microbiome and immunity. Cancer Discov.

[6] Hayase E, Hayase T, Mukherjee A (2024). Bacteroides ovatus alleviates dysbiotic microbiota-induced graft-versus-host disease. Cell Host Microbe.

[7] Koskenvuo L, Lunkka P, Varpe P (2024). Morbidity after mechanical bowel preparation and oral antibiotics prior to rectal resection: the MOBILE2 randomized clinical trial. JAMA Surg.

[8] Sharma DK, Ramadass B, Callary SA (2024). The effect of prebiotic fibre on the gut microbiome and surgical outcomes in patients with prosthetic joint infection (PENGUIN) - study protocol for a randomised, double-blind, placebo-controlled trial (ACTRN12623001273673). Nutr J.

[9] Wang M, Rousseau B, Qiu K (2024). Killing tumor-associated bacteria with a liposomal antibiotic generates neoantigens that induce anti-tumor immune responses. Nat Biotechnol.

[10] Salvati F, Catania F, Murri R, Fantoni M, Torti C (2024). Clostridioides difficile infection: an update. Infez Med.

[11] Weaver L, Troester A, Jahansouz C (2024). The impact of surgical bowel preparation on the microbiome in colon and rectal surgery. Antibiotics (Basel).

[12] Shaikh FY, Lee S, White JR (2024). Fecal microbiome composition correlates with pathologic complete response in patients with operable esophageal cancer treated with combined chemoradiotherapy and immunotherapy. Cancers (Basel).

[13] Del Chierico F, Piazzesi A, Fiscarelli EV (2024). Changes in the pharyngeal and nasal microbiota in pediatric patients with adenotonsillar hypertrophy. Microbiol Spectr.

[14] Finazzi Agrò E, Rosato E, Wagg A (2024). How do we make progress in phenotyping patients with LUT such as OAB and underactive detrusor, including using urine markers and microbiome data, in order to personalize therapy? ICI-RS 2023: Part 1. Neurourol Urodyn.

[15] Gorecka-Mazur A, Krygowska-Wajs A, Furgala A (2024). Associations between gut microbiota characteristics and non-motor symptoms following pharmacological and surgical treatments in Parkinson's disease patients. Neurogastroenterol Motil.

[16] Liang X, Zhu Y, Bu Y (2024). Microbiome and metabolome analysis in smoking and non-smoking pancreatic ductal adenocarcinoma patients. BMC Microbiol.

[17] Seo H, Park JY, You HS, Kim BJ, Kim JG (2025). Comparative changes in fecal microbiome after endoscopic resection and surgical resection in gastric cancer patients. J Pers Med.

[18] Zambouri A (2007). Preoperative evaluation and preparation for anesthesia and surgery. Hippokratia.

[19] Yang Y, Xia Y, Chen H (2016). The effect of perioperative probiotics treatment for colorectal cancer: short-term outcomes of a randomized controlled trial. Oncotarget.

[20] Flesch AT, Tonial ST, Contu PC, Damin DC (2017). Perioperative synbiotics administration decreases postoperative infections in patients with colorectal cancer: a randomized, double-blind clinical trial. Rev Col Bras Cir.

[21] Grąt M, Wronka KM, Lewandowski Z (2017). Effects of continuous use of probiotics before liver transplantation: a randomized, double-blind, placebo-controlled trial. Clin Nutr.

[22] Kotzampassi K, Stavrou G, Damoraki G, Georgitsi M, Basdanis G, Tsaousi G, Giamarellos-Bourboulis EJ (2015). A four-probiotics regimen reduces postoperative complications after colorectal surgery: a randomized, double-blind, placebo-controlled study. World J Surg.

[23] Rayes N, Seehofer D, Neuhaus P (2009). Prebiotics, probiotics, synbiotics in surgery—are they only trendy, truly effective or even dangerous?. Langenbecks Arch Surg.

[24] Sze MA, Baxter NT, Ruffin MT IV, Rogers MA, Schloss PD (2017). Normalization of the microbiota in patients after treatment for colonic lesions. Microbiome.

[25] Bajramagic S, Hodzic E, Mulabdic A, Holjan S, Smajlovic SV, Rovcanin A (2019). Usage of probiotics and its clinical significance at surgically treated patients sufferig from colorectal carcinoma. Med Arch.

[26] Komatsu S, Sakamoto E, Norimizu S, Shingu Y, Asahara T, Nomoto K, Nagino M (2016). Efficacy of perioperative synbiotics treatment for the prevention of surgical site infection after laparoscopic colorectal surgery: a randomized controlled trial. Surg Today.

[27] McNaught CE, Woodcock NP, Anderson AD, MacFie J (2005). A prospective randomised trial of probiotics in critically ill patients. Clin Nutr.

[28] Sommacal HM, Bersch VP, Vitola SP, Osvaldt AB (2015). Perioperative synbiotics decrease postoperative complications in periampullary neoplasms: a randomized, double-blind clinical trial. Nutr Cancer.

[29] Polakowski CB, Kato M, Preti VB, Schieferdecker ME, Ligocki Campos AC (2019). Impact of the preoperative use of synbiotics in colorectal cancer patients: a prospective, randomized, double-blind, placebo-controlled study. Nutrition.

[30] Liu Z, Li C, Huang M (2015). Positive regulatory effects of perioperative probiotic treatment on postoperative liver complications after colorectal liver metastases surgery: a double-center and double-blind randomized clinical trial. BMC Gastroenterol.

[31] Rayes N, Hansen S, Seehofer D, Müller AR, Serke S, Bengmark S, Neuhaus P (2002). Early enteral supply of fiber and Lactobacilli versus conventional nutrition: a controlled trial in patients with major abdominal surgery. Nutrition.

[32] Deng X, Li Z, Li G, Li B, Jin X, Lyu G (2018). Comparison of microbiota in patients treated by surgery or chemotherapy by 16S rRNA sequencing reveals potential biomarkers for colorectal cancer therapy. Front Microbiol.

[33] Rayes N, Seehofer D, Theruvath T (2007). Effect of enteral nutrition and synbiotics on bacterial infection rates after pylorus-preserving pancreatoduodenectomy: a randomized, double-blind trial. Ann Surg.

[34] Park IJ, Lee JH, Kye BH (2020). Effects of PrObiotics on the Symptoms and Surgical ouTComes after Anterior REsection of Colon Cancer (POSTCARE): a randomized, double-blind, placebo-controlled trial. J Clin Med.

[35] Fang X, Vázquez-Baeza Y, Elijah E (2021). Gastrointestinal surgery for inflammatory bowel disease persistently lowers microbiome and metabolome diversity. Inflamm Bowel Dis.

